# A Reproducible Benchmark for Leakage-Resistant Machine Learning on Multi-Site Brain Connectivity Graphs

**DOI:** 10.3390/diagnostics16203312

**Published:** 2026-10-12

**Authors:** Abu Ubaida, Khurram Zeeshan Haider, Salabat Khan, Celal Alagoz, Farhan Aadil

**Affiliations:** 1 Center of Data Science, Government College University Faisalabad, Faisalabad 38000, Pakistan; abuubaida.202202778@gcuf.edu.pk; 2 Department of Software Engineering, Government College University Faisalabad, Faisalabad 38000, Pakistan; khurram.zeeshan@gcuf.edu.pk; 3 Faculty of Computer Studies, Arab Open University, A’ali P.O. Box 18211, Bahrain; 4 Department of Computer Engineering, Sivas University of Science and Technology, Sivas 58000, Türkiye; alagozc@sivas.edu.tr (C.A.); farhan.aadil@sivas.edu.tr (F.A.)

**Keywords:** autism spectrum disorder, functional magnetic resonance imaging, graph neural networks, data leakage, leave-one-site-out validation, differential privacy, membership inference, federated learning, reproducibility

## Abstract

**Background/Objectives:** Machine-learning studies of autism spectrum disorder from resting-state functional magnetic resonance imaging routinely report near-perfect accuracy, yet the models rarely transfer to an unseen scanner. We audited a pipeline of the kind this literature builds, which reported an accuracy of 0.999, and traced that number to five data-leakage pathways. **Methods:** Under a nested leave-one-site-out protocol on 871 participants from the Autism Brain Imaging Data Exchange, in which the held-out site influenced nothing but model selection, the threshold, the combiner, or the site correction, we closed every pathway, priced each by reopening it, and validated on a locked external cohort. **Results:** With all five pathways closed, the graph attention network reached an area under the curve of 0.648 and did not outperform a support vector machine on the same connectivity (0.679; *p* = 0.009). Decision-level fusion added nothing detectable (0.682, *p* = 0.72). A network identified its training participants better than it detected autism (0.710 versus 0.610); both questionnaire benchmarks proved label-tautological, and performance held on the external cohort. **Conclusions:** Reported gains in this literature should be read as claims about evaluation protocols until shown otherwise on an untouched acquisition site; all predictions, models, and code are released.

## 1. Introduction

Autism spectrum disorder is diagnosed by structured behavioral observation, a process that requires trained clinicians and hours of assessment. The prospect of an objective marker derived from a ten-minute resting-state functional magnetic resonance imaging scan has therefore motivated a large literature, and the release of the Autism Brain Imaging Data Exchange [1] gave it a common substrate. Reported performance has risen steadily: the first multi-site analysis reported approximately sixty percent accuracy; recent graph-learning and transformer models report areas under the receiver operating characteristic curve between 0.70 and 0.80 [2], and studies that add questionnaire data report accuracies of ninety-five to one hundred percent.

That trajectory sits awkwardly beside two established facts. First, reliable brain-wide association effects require sample sizes in the thousands [3], and this cohort has fewer than nine hundred usable participants. Second, when the problem was posed as a blind community challenge, the best submissions reached an area under the curve of about 0.80 on the private set and fell to 0.72 on an external site added after the challenge closed [4]. The distance between a blind challenge and the published literature is a distance in evaluation, not in biology.

Leakage is the standard name for that distance: the family of mechanisms by which information about the evaluation data reaches the model before it is evaluated. It inflates reported performance across the machine-learning-based sciences [5], in medical imaging [6], and, most directly, in connectome-based predictive models, where several common forms of leakage each raise apparent accuracy [7]. Neuroimaging is unusually exposed because the acquisition site is itself predictive [8].

This paper begins from an audit rather than a method, and the object of that audit is a pipeline built in the manner this literature treats as standard a graph neural network on brain connectivity, two questionnaire screening sites, a differentially private decision-level combiner and a membership-inference evaluation which reported an accuracy of 0.999. Reading its code rather than its output revealed five leakage pathways (Figure 1): the questionnaire label is a threshold of its own items (L1); the held-out site selected the checkpoint (L2); the fusion combiner was fitted on the participants it was scored on (L3); the probability stream analyzed for privacy was drawn from the label (L4); and the site correction was fitted across all sites, the held-out one included (L5). Closing all five leaves the pipeline this paper studies, and Section 3 prices each pathway by reopening it.

We use “leakage” in the broad sense established in this literature: any pathway, beyond classical train/test row overlap, by which information about the held-out site reaches the model, its selection, or its evaluation before that site is scored. L1 and L5 are leakage in this sense even though neither is row overlap: L1 lets the label be recovered from the input by construction, which inflates apparent performance in exactly the way row overlap does; L5 lets the held-out site’s own scans shape the preprocessing applied to it. Section 4 returns to this terminology and to why each pathway is treated as a leak rather than a legitimate design choice.

The findings that follow are mostly negative. They are reported because a negative result obtained under a protocol that can be checked is worth more than a positive one obtained under a protocol that cannot.

### Related Work

Representing each participant as a connectivity graph fits functional imaging naturally, and the architectures have followed one another quickly: interpretable region-selecting graph networks [9], transformers over the connectome [2], causality-inspired graph networks, and domain-adaptive variants aimed at the multi-site problem. The most recent multi-site and multi-center work continues in this direction, with classifiers spanning both releases of the Autism Brain Imaging Data Exchange [10] and multi-view graph structure learning across atlases and centers [11]. Reported areas under the curve cluster between 0.70 and 0.80; few of these evaluations hold out an entire acquisition site.

The mechanisms by which a validation protocol flattens a model are well catalogued: optimism from small samples, and leakage through feature selection, whole-dataset confound correction, or a preprocessing transform fitted before splitting, which in connectome-based prediction inflates performance by amounts comparable to the effects reported [7,12]. Site correction is the standard remedy for scanner variance [13], but it is also a route through which the held-out site crosses the split [14,15]. Formal privacy comes from differentially private training [16] under Rényi accounting [17], and its worth is tested by attack [18,19]; the privacy–accuracy frontier in medical imaging, and its uneven burden, are active areas of study [20,21,22]. That classical models remain competitive with deep architectures on data of this size is well established [23].

## 2. Materials and Methods

### 2.1. Imaging Cohort

All imaging analyses use the first release of the Autism Brain Imaging Data Exchange [1], from the preprocessed release produced with the Configurable Pipeline for the Analysis of Connectomes [24], averaged within the 116 regions of the Automated Anatomical Labeling atlas [25]. After quality control, 871 participants remain (403 with a clinical diagnosis of autism spectrum disorder and 468 typically developing); the subset is also used by Abraham and colleagues [8]. The cohort is treated as 17 acquisition sites (three same-scanner sub-acquisitions pooled). Table 1 summarizes them.

### 2.2. Connectivity, Site Correction and Graph Construction

For each participant, the connectivity between regions *i* and *j* is the Pearson correlation of their regional time courses,

(1) $$c_{ij}=\frac{\sum_{t=1}^{T}\left(x_{it}-{\bar{x}}_{i}\right)\left(x_{jt}-{\bar{x}}_{j}\right)}{\sqrt{\sum_{t}^{T}{\left(x_{it}-{\bar{x}}_{i}\right)}^{2}\sum_{t}^{T}{\left(x_{jt}-{\bar{x}}_{j}\right)}^{2}}}$$

Because the acquisition site dominates the variance of these values, each entry is standardized within its site and returned to the common scale,

(2) $${\tilde{c}}_{ij}=\frac{c_{ij}-\mu_{s}}{\sigma_{s}}+\mu_{global}$$

where $\mu_{s}$ and $\sigma_{s}$ are the mean and standard deviation of that connection across site s. Where the correction is fitted matters more than what it is. Table 2 summarizes this and the other four leakage pathways audited in this paper. The audited pipeline, like most of this literature, fitted it once across all seventeen sites, so the held-out site helped define the correction applied to it. Here it is refitted inside every fold on the training sites, in two settings:

- Strict: the held-out site receives no correction; its statistics cannot be estimated without its data, so its connectivity passes through unchanged while the training sites are standardized. This is the deployment condition for an unseen scanner, and all headline numbers use it.
- Transductively, the held-out site’s own unlabeled scans set its statistics, as almost every multi-site study does. No diagnostic label is used; Section 3.5 measures what it is worth.

The graph retains the K = 20 connections of largest magnitude per region, symmetrized; the edge weight is the corrected connectivity value. Each region’s feature vector concatenates its full corrected connectivity row with eight time-course statistics (mean, standard deviation, variance, skewness, kurtosis, minimum, maximum, range), giving 124 features, standardized across the regions of the same participant. Figure 2 shows the whole pipeline and the network architecture.

### 2.3. Graph Attention Network and Objective

All experiments were implemented in Python 3.11 using PyTorch (≥2.1.0) and PyTorch Geometric (≥2.4.0) for the graph neural network, scikit-learn (≥1.3.0) for the classical reference models, and nilearn (≥0.10.3) for imaging data handling. Three graph attention layers [26] propagate information along the retained edges; the attention coefficient of region j for region i weighs the corrected edge value $e_{ij}$, which the audited implementation discarded. Representations are updated over four heads with residual connections and pooled by concatenating mean and maximum pooling. Class imbalance is handled with a class-weighted focal loss [27] (γ = 2), optimized with decoupled weight decay, cosine annealing, and gradient clipping; the coefficient is $e_{ij}.$

A graph attention network was chosen over a plain graph convolutional network because attention weights over edges are directly interpretable as the relative importance of each retained connection, which matters for auditing what the network actually uses; over a full graph transformer because the sparsified K = 20 graph already discards most possible edges, so global attention across all region pairs adds parameters without adding structure the sparsification has not already removed. This choice does not affect the paper’s central negative finding, which concerns evaluation protocol rather than architecture: the same leakage pathways would inflate any graph-based model built on this pipeline.

(3) $$\alpha_{ij}=\frac{\exp\left(\mathrm{LeakyReLU}a^{⊤}\;[Wh_{i}\;\|\;Wh_{j}\;\|\;We_{ij}]\right)}{\sum_{k\in N\left(i\right)}\exp\left(\mathrm{LeakyReLU}a^{⊤}\;[Wh_{i}\;\|\;Wh_{k}\;\|\;We_{ik}]\right)}$$

### 2.4. Reference Models

Three classical models are trained on the upper triangle of the sparsified connectivity matrix, a vector of 6670 values of which 1550 (23 percent) are non-zero: a radial-basis-function support vector machine, a random forest, and a penalized logistic regression. The graph network’s node features carry each region’s full dense connectivity row, so the classical models are given strictly less information, which strengthens the comparison of Section 3.1. A fourth model uses demographic covariates only (age, sex, three intelligence quotients, handedness, motion, eye status) and quantifies how much of any fused performance is demography rather than imaging [23].

A fourth graph model, distinguished from the graph attention network of Section 2.3 by region-aware components in the style of BrainGNN, is trained under the identical strict protocol. Two graph convolution blocks, each conditioned on a learned per-region-of-interest embedding rather than treating every region interchangeably, are separated by a learned top-k node-selection layer that keeps the half of the regions the network scores as most informative before the second convolution, so both which regions matter and how they are convolved depend on region identity. It uses the same site correction, graph construction, training objective and leave-one-site-out folds as every other model in Section 3.1.

### 2.5. Decision-Level Fusion

A learned weighted mean combines the per-participant probabilities,

(4) $${p_{s}}^{fused}=\mathrm{clip}\sum_{k=1}^{K}w_{k}p_{sk},0,1$$

The weights and the decision threshold are refitted seventeen times, once per held-out site, on the sixteen remaining sites; a combiner fitted on the participants it is later scored on is a leak, and the size of that leak is quantified in Section 3.3.

### 2.6. Differential Privacy

Two mechanisms are evaluated. First, only the combiner gradient is privatized: the mini-batch mean gradient is clipped to norm C and perturbed [16],

(5) $$\tilde{g}=\frac{\bar{g}}{\max\left(1,\|\bar{g}\|\;/\;C\right)}+N\left(0,\frac{\sigma^{2}C^{2}}{B^{2}}I\right)$$

Second, the per-participant probability each site transmits is itself perturbed what a site must do to protect the transmitted quantity. Under the sampled Gaussian mechanism the Rényi divergence accumulates as [17],

(6) $$\rho_{\alpha }\leq T\cdot \frac{1}{\alpha -1}\log\left(1+q^{2}\left(\exp\left(\frac{\alpha \left(\alpha -1\right)}{2\sigma^{2}}\right)-1\right)\right)$$

And is converted to the standard formulation [17] by

(7) $$\varepsilon \left(\delta \right)=\min._{\alpha }\left(\rho_{\alpha }+\frac{\log\left(1/\delta \right)}{\alpha -1}\right)$$

Over integer orders α ∈ [2, 256] and δ = 10^−5^. Two caveats: the accountant assumes per-example clipping, while the implementation clips the batch mean, so the budgets are illustrative rather than certified, and it models only the gradient mechanism, whereas perturbing the transmitted probabilities is an additional release (Section 3.4).

### 2.7. Membership Inference

A graph network is trained on a stratified half of the cohort. A confidence-thresholding attack [18] and a ten-shadow-model attack [19] use only the transmitted probabilities; the attacked network’s own diagnostic performance is measured on its own held-out half, so the two quantities compared in Section 3.7 come from the same model.

### 2.8. Federated Training

The seventeen sites are treated as seventeen silos; over thirty rounds, each trains locally for two epochs and returns its parameters, averaged by silo size [28]. In the private variant, each silo clips and perturbs its update; the aggregate noise multiplier is σ/√K ≈ 0.20, below the nominal σ = 0.8, so the reported budget is optimistic.

### 2.9. Evaluation Protocol and Statistics

One acquisition site is held out from every training, selection, and thresholding decision. Within the remaining sixteen, four further sites form a development set on which the configuration is chosen among four candidates, and a stratified twelve percent of the training participants provides the early-stopping signal. The winner is retrained on all sixteen with three seeds and applied once to the held-out site. Discrimination is the area under the curve; operating points use balanced accuracy and the Matthews correlation coefficient (MCC) [29]. Intervals are 10,000-sample bootstraps; comparisons use the paired bootstrap of the area-under-the-curve difference, McNemar’s test, and a one-sided site-level Wilcoxon test.

The protocol amounts to 136 configuration-selection trainings, 102 final trainings, and 11 for the membership-inference audit, plus 16,320 short local trainings for the federated experiments; it completes in about one hour on a single graphics processing unit.

The full protocol (136 configuration-selection trainings, 102 final trainings, 11 membership-inference runs and 16,320 short federated-learning updates) completes in approximately one hour on a single graphics processing unit, so the nested, nothing-touches-the-held-out-site design used throughout this paper is computationally inexpensive relative to the scale of the graphs and cohort involved, and is practical to adopt as a standard.

## 3. Results

### 3.1. A Graph Network Does Not Outperform a Kernel Machine

Table 3 reports every model under the strict protocol. The graph attention network reaches an area under the curve of 0.648 (0.612–0.684); the support vector machine, given the sparsified quarter of the same connectivity, reaches 0.679 (0.644–0.714) (Figure 3). The evidence is mixed and reported as such: the kernel machine classifies more participants correctly (McNemar 157 vs. 113 discordant, *p* = 0.009) and is better at thirteen of seventeen sites (Wilcoxon *p* = 0.015), while its advantage in area under the curve is 0.031 with a 95% interval of −0.002 to 0.065 (*p* = 0.063) that contains zero. The claim this paper makes is the negative one: after a nested search on data the held-out site never touched, the graph network does not outperform a kernel machine handed strictly less information.

In its audited form with the held-out site selecting the checkpoint and the edge weights discarded, the same architecture predicted a single class for almost every participant once that selection was removed. Restoring the edge weights and choosing the configuration on development sites lifted it to 0.578; refitting the site correction inside the fold lifted it again to 0.648. Global correction was harming the graph network because it distorted the edge weights the attention mechanism reads.

A region-aware architecture does not change this picture. The BrainGNN-style network reaches an area under the curve of 0.675 (0.640–0.711), between the attention network and the kernel machine and not distinguishable from either (kernel machine: Δ0.004, 95% CI −0.034 to +0.027, *p* = 0.823; attention network: Δ0.027, 95% CI −0.004 to +0.059, *p* = 0.087). Under the same nested, held-out-site-blind protocol, region-aware pooling narrows the gap to the kernel machine without closing it, evidence that the negative result concerns the evaluation protocol rather than one architecture’s specific limitations.

### 3.2. The Comparison Site by Site

Across the seventeen held-out sites, the graph network attains a mean area under the curve of 0.653 against 0.686 for the kernel machine (Wilcoxon *p* = 0.015); the kernel machine is better at 13 of seventeen sites, and the graph network falls below chance at 2 (Table 4, Figure 4).

### 3.3. Decision-Level Fusion Adds Nothing Detectable

Fusing the kernel machine with the demographic model raises the area under the curve to 0.682; the paired difference over the kernel machine is 0.003 (*p* = 0.72), the interval containing zero. Adding the graph network lowers the Matthews correlation coefficient and earns a least-squares weight of only +0.07 against the kernel machine’s +0.61; the combiner all but ignores it. Fitting the same combiner in-sample rather than out-of-site raises its apparent area under the curve from 0.682 to 0.697 (Table 5); the gap is leakage, not performance.

### 3.4. Pricing the Pathways

Each pathway can be reopened, so each can be priced (Table 6, Figure 5). The audited pipeline’s headline balanced accuracy 0.999 is reproduced exactly by re-running its released code. Removing the two tautological questionnaires costs −0.362: they supplied almost all of the apparent performance. Real out-of-fold outputs and honest model selection cost a further −0.080. Refitting the combiner out of site and reporting the best honest system returns +0.089 (a change of reported system, not a closed leak). Fitting the site correction in-fold costs −0.013, leaving 0.632.

### 3.5. The Value of the Site Correction

Almost every multi-site study fits its site correction across the whole cohort, so the held-out site’s own scans help define the correction applied to it. Table 7 and Figure 6 report both settings on identical folds. The transductive setting is worth +0.024 area under the curve to the fusion (*p* = 0.026) and +0.031 to the random forest (*p* = 0.019); for the kernel machine the gain is +0.020 with an interval containing zero (*p* = 0.053), and the graph network gains nothing (−0.002). The effect is real but model-dependent, significant for two of five imaging models; where significant, it is worth two to three points, more than this literature routinely reports between architectures. Two cautions cut against our interest: the strict setting applies no correction to the held-out site, so the difference bounds the value of transductive information from above; and the configuration is re-selected per setting, differing at 4 of seventeen folds, so the graph network’s row confounds correction with an architecture change. The phenotype model, which never sees imaging, is unchanged as a control.

### 3.6. Differential Privacy Is Cheap Only Where It Protects Almost Nothing

Noise on the two combiner weights leaves utility unchanged (0.703 to 0.703 at ε ≈ 6.4): the combiner has almost no capacity to lose. But when the transmitted probabilities are perturbed as well, at the same nominal noise, the fused area under the curve falls from 0.690 to 0.519, which is chance (Table 8, Figure 7). Both mechanisms are reported at the same ε; only one protects the transmitted data. This experiment and the federated one predate the in-fold correction and use data corrected across all sites, so their numbers are upper bounds; both are negative results.

### 3.7. A Better Membership Oracle than a Diagnostic Instrument

A network trained on a stratified half classifies its own training participants 30.5 points more accurately than the half it never saw. Confidence thresholding recovers membership at 0.715; a ten-shadow attack at 0.710. On the same held-out half, its diagnostic area under the curve is 0.610: the network is a more reliable oracle for training-set membership than for the disorder (Table 9, Figure 8). Perturbing the probabilities cuts the attacks to 0.539 and 0.526, but Section 3.6 shows what that costs.

### 3.8. Federated Averaging

Federated averaging across the seventeen silos gives a mean area under the curve of 0.630, 0.023 below the 0.653 of the centrally trained network on the same basis; federation is competitive but not superior. Noisy updates reduce it to a 0.500 chance, while the accountant returns ε ≈ 58, which is no guarantee (Table 10, Figure 9).

### 3.9. The Screening Questionnaires Label Themselves

The two questionnaire datasets, used as two of the audited pipeline’s three sites, are not classification problems. In both, the published label is recovered exactly in every record by thresholding the sum of the ten items: at four for the toddler checklist (1054 records) and seven for the adult self-report (704 records) (Table 11, Figure 10). A model trained on those items re-derives an arithmetic rule it was handed, and the ninety-five to one hundred percent accuracies reported on them measure that fact and nothing else. We exclude both from every model.

### 3.10. External-Cohort Validation

A stricter test holds out several sites at once. The three largest sites, NYU, UM, and USM, 359 participants were locked away and used for nothing; every model was trained, selected, and thresholded on the other fourteen (512 participants) and evaluated once on the locked three. Performance did not fall; the kernel machine reached 0.738 against 0.679 under leave-one-site-out, the graph network 0.706 against 0.648, and the fusion 0.727 (Table 12, Figure 11). The ordering is unchanged, so the negative result is not an artifact of the leave-one-site-out protocol. A truly independent cohort matched to this pipeline was unavailable, because the second release does not distribute the atlas time courses this pipeline uses.

The locked cohort (NYU, UM, USM) differs from the training sites in scanner vendor and acquisition protocol but is drawn from the same ABIDE-I release, so age and sex distributions are comparable to the training sites (Table 1), and no additional harmonization was applied beyond the site correction described in Section 2.2. This is a within-release, cross-scanner test of domain shift rather than a fully independent cohort; the Limitations section notes this limitation explicitly.

## 4. Discussion

The central result is a subtraction, performed one term at a time. A pipeline that reported a balanced accuracy of 0.999 supports, once five leakage pathways are closed, 0.632. The difference is an evaluation artifact, not a modeling failure. Each pathway is individually mundane and documented before what is instructive is how naturally they compose: a tautological label supplies an unbeatable site, an in-sample combiner converts it into a fused number, test-set model selection shields the imaging branch, and a label-derived probability stream makes the privacy analysis appear sound. No step felt like a shortcut in isolation.

That the kernel machine matches the graph network is unsurprising: the connectivity vector already contains the pairwise interactions message passing propagates, and the graph prior buys structure at the cost of parameters on a cohort where the ceiling on any brain-wide association is low [3]. Our contribution is not the direction of the gap but its character: it survives a nested search on data the held-out site never touched, and the graph network, with its extra structure and the dense matrix the kernel machine never sees, buys nothing. The privacy findings are equally plain: a guarantee is a statement about a released quantity, and noise on two combiner weights protects two numbers, not the per-participant probabilities transmitted to compute them. When the quantity that must be protected is protected, the task does not survive it. The membership result sharpens the point: the network identifies its training participants at 0.710 while identifying the disorder at 0.610. Finally, the questionnaire result is a caution about cheap benchmarks whose label is an arithmetic function of the input; the remedy is a habit: before reporting a classifier’s accuracy, check whether the label can be computed from the features by hand.

These results have three practical implications. For clinical translation, none of the models here reaches a performance level that would support diagnostic use, so claims of near-perfect accuracy in this literature should be treated as evaluation artifacts rather than progress toward a deployable marker. For biomarker development, the finding that a kernel machine matches a graph network under a protocol the held-out site never touches suggests that architectural complexity is not the limiting factor at this sample size; effort is better spent on larger, better-harmonized multi-site cohorts than on new network designs. For future multi-center neuroimaging studies, we recommend as a minimum: fitting any site correction inside each cross-validation fold, selecting models and thresholds without reference to the held-out site, and reporting performance on a locked external cohort before claiming generalization. Beyond autism classification, the leakage-resistant evaluation protocol proposed here is disorder-agnostic and applies directly to any multi-site classification problem built on brain connectivity, including other psychiatric and neurological conditions.

Three of the five pathways warrant a note on scope. L1 is not train/test overlap; it is a task-design flaw in which the label is a deterministic function of the input, and we flag it because it inflates apparent performance by the same mechanism as overlap does, not because it is leakage in the classical sense. L4 is a property of the specific audited implementation rather than a claim that every autism neuroimaging pipeline transmits label-derived probabilities; it is reported as a concrete instance of a general risk: that a privacy analysis can certify the wrong released quantity, which is why Section 3.6 states it as a caution rather than a field-wide indictment. L5, using the held-out site’s own unlabeled scans to fit its site correction, is standard practice in this literature, and we do not claim it is illegitimate; we call it a leakage pathway because Section 3.5 shows it is not free: it moves the fused and random-forest area under the curve by two to three points, a magnitude comparable to differences this literature reports between architectures. Reporting it under the strict, no-correction-from-the-held-out-site setting, and quantifying the transductive gain separately, is this paper’s recommendation, not an assertion that the transductive setting is invalid.

### Limitations

- A single data-collection effort; the locked external cohort tests generalization across scanners within one release, but a truly independent second cohort matched to this pipeline was unavailable. The cohort is predominantly male and Western.
- The privacy and federated experiments were not repeated under the in-fold correction, so their numbers are upper bounds; both are negative results, so the residual optimism works against them.
- The differential-privacy budgets are illustrative rather than certified; the accountant assumes per-example clipping, while the code clips the batch mean, but every such error acts against the privacy claims.
- This is a research benchmark, not a clinical instrument, and the demographic model is a confound rather than a biomarker (area under the curve 0.615); no deployment claim is warranted.
- The cohort is drawn entirely from ABIDE-I; whether these conclusions transfer to ABIDE-II, UK Biobank, the Human Connectome Project, or other multi-site neuroimaging resources is not established here and should be tested directly rather than assumed.
- A single preprocessing pipeline (C-PAC, AAL atlas, Pearson connectivity) is evaluated throughout; ICA-based pipelines, alternative atlases, and dynamic functional-connectivity measures may shift absolute performance even if the leakage pathways identified here still apply.
- The audit is conducted on a single disorder and imaging cohort (ABIDE-I autism data) because the inflated claim it audits (balanced accuracy 0.999) was made on exactly this dataset; pricing the five leakage pathways required auditing the pipeline that exhibited them. Whether pathways of the same kind, and of similar cost, recur in other disorders and cohorts, for example depression cohorts such as REST-meta-MDD, is a natural extension we did not attempt here and recommend as future work rather than folding it into an already five-pathway audit.

## 5. Conclusions

We audited a multi-instrument, privacy-preserving autism screening pipeline, found five leakage pathways behind its reported balanced accuracy of 0.999, closed them all, and priced each. A graph attention network on brain connectivity reaches an area under the curve of 0.648 and does not outperform a support vector machine given strictly less of the same connectivity (0.679). Decision-level fusion adds nothing detectable; differential privacy is inexpensive only where it protects almost nothing; the same network that detects autism at 0.610 reveals its training participants at 0.710; and both questionnaire benchmarks are label-tautological. This is a methodological contribution: an audit and a leakage-resistant evaluation protocol, not a claim that any classifier here is superior for autism screening. None of this is an artifact of the protocol: on a locked external cohort of 359 participants, every figure is maintained. The next paper in this literature would be more useful reporting a lower number under a protocol that can be checked than a higher one under a protocol that cannot; all predictions, models, and code are released so that the checking is possible.

## Figures and Tables

**Figure 1:**
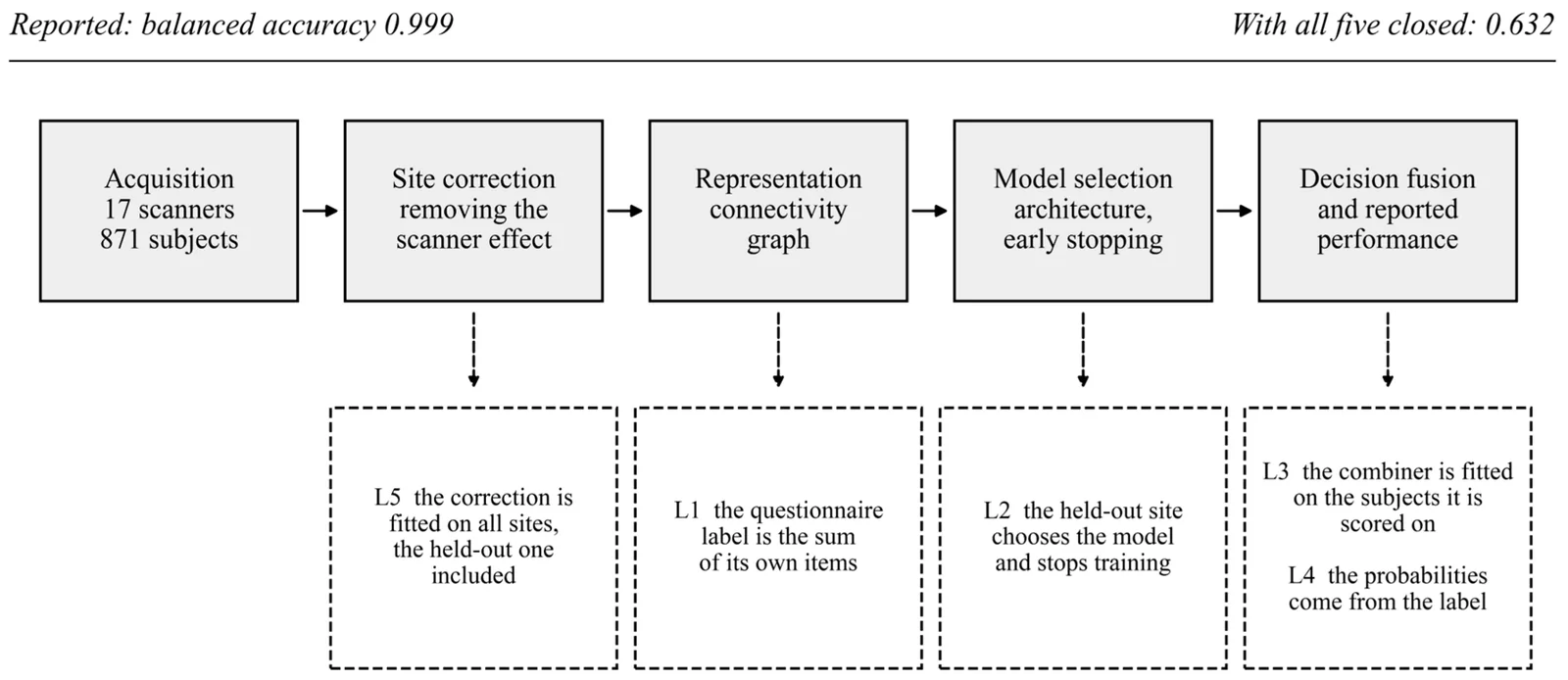
Where an autism screening evaluation loses its meaning. Beneath each stage of the shared pipeline is a leakage pathway found in the audited implementation (L1–L5). With all five closed, the same data support a balanced accuracy of 0.632 rather than the reported 0.999.

**Figure 2:**
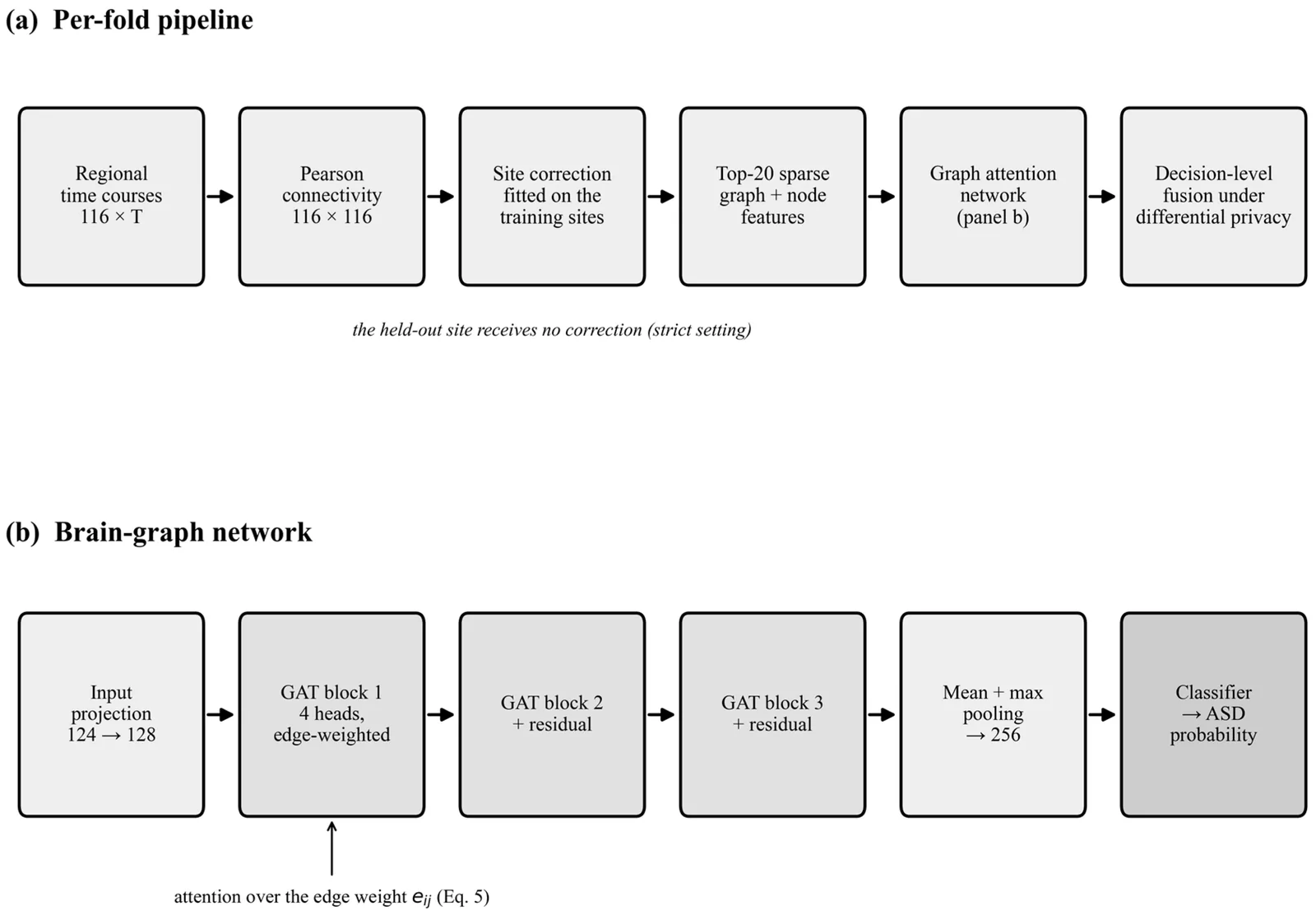
The method. (**a**) The per-fold pipeline: regional time courses become a connectivity matrix, standardized by a site correction fitted on the training sites, then the sparse graph, the graph attention network, and the differentially private decision-level fusion. (**b**) The graph attention network: an input projection, three edge-weighted attention blocks with residual connections, pooling, and a classifier.

**Figure 3:**
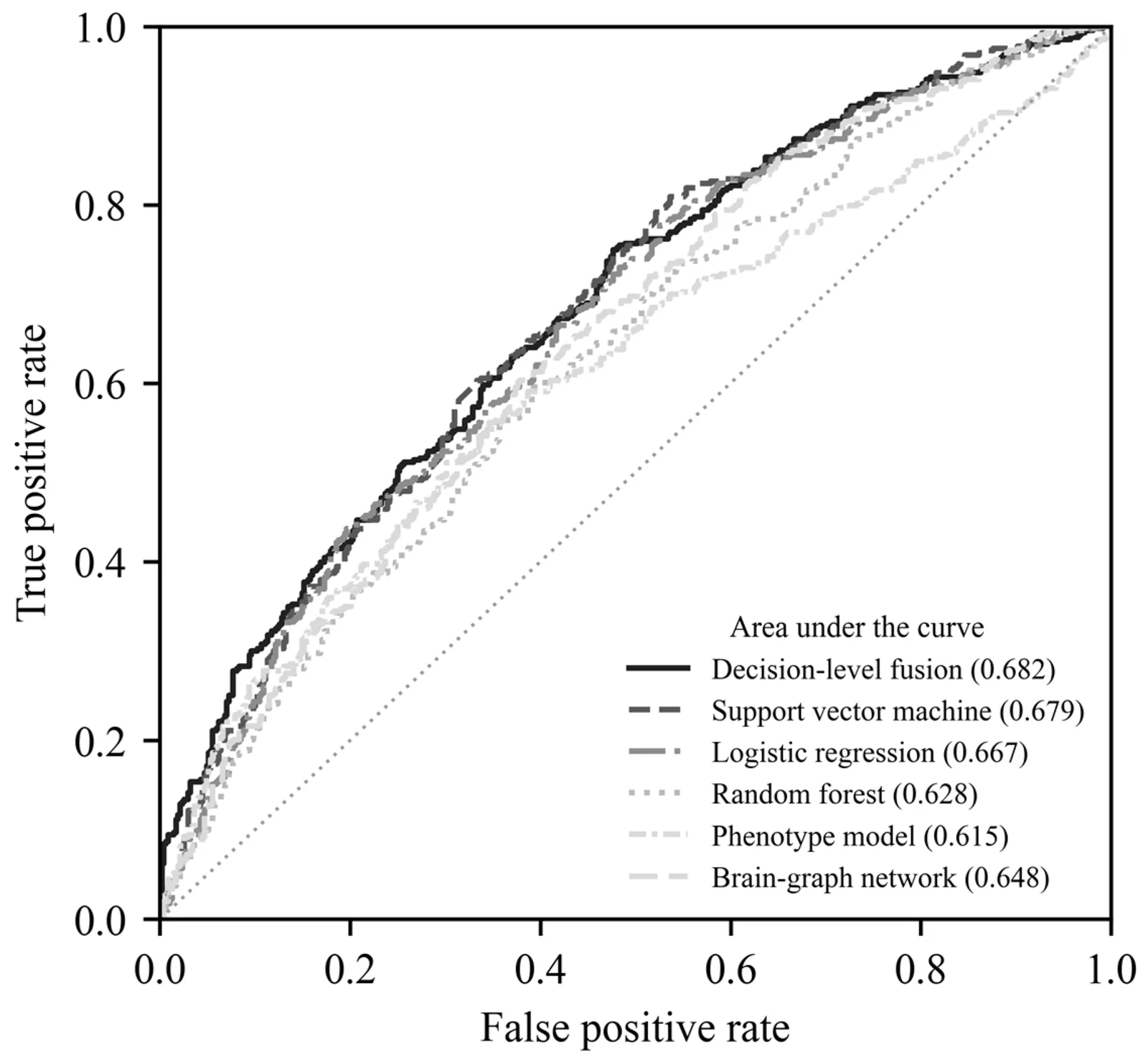
Receiver operating characteristic curves, pooled over all 871 participants. The brain-graph network is the weakest model despite receiving the dense connectivity matrix; the kernel machine is the strongest single model. All six models are shown: decision-level fusion, support vector machine, logistic regression, random forest, phenotype model and brain-graph network, each labelled with its pooled area under the curve; “single model” in the sentence above distinguishes the five individual classifiers from the fusion of them.

**Figure 4:**
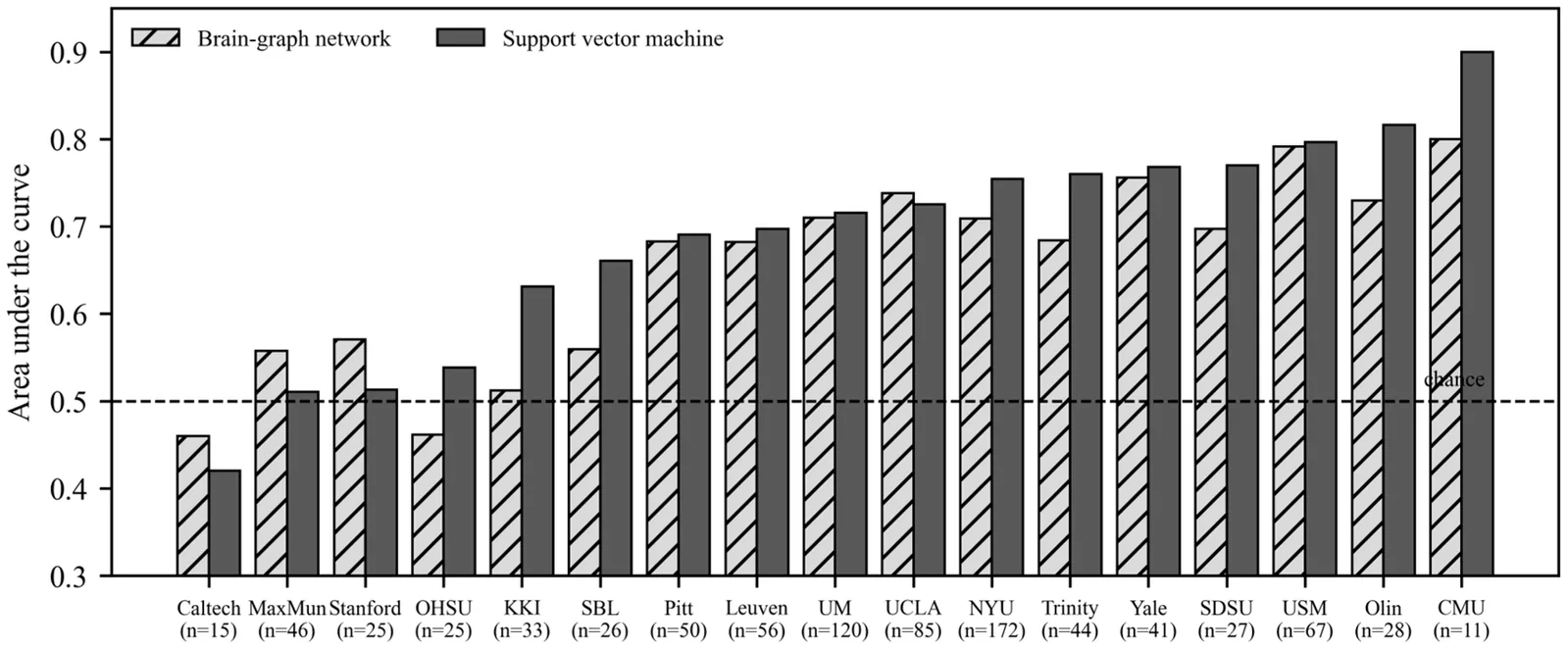
Area under the curve per held-out site, ordered by the kernel machine. The brain-graph network (hatched) is below the kernel machine at 13 of seventeen sites and below chance at 2. Sites are ordered left to right by the kernel machine’s area under the curve at that site, so a site where the hatched bar exceeds the solid bar is one of the four sites at which the brain-graph network wins.

**Figure 5:**
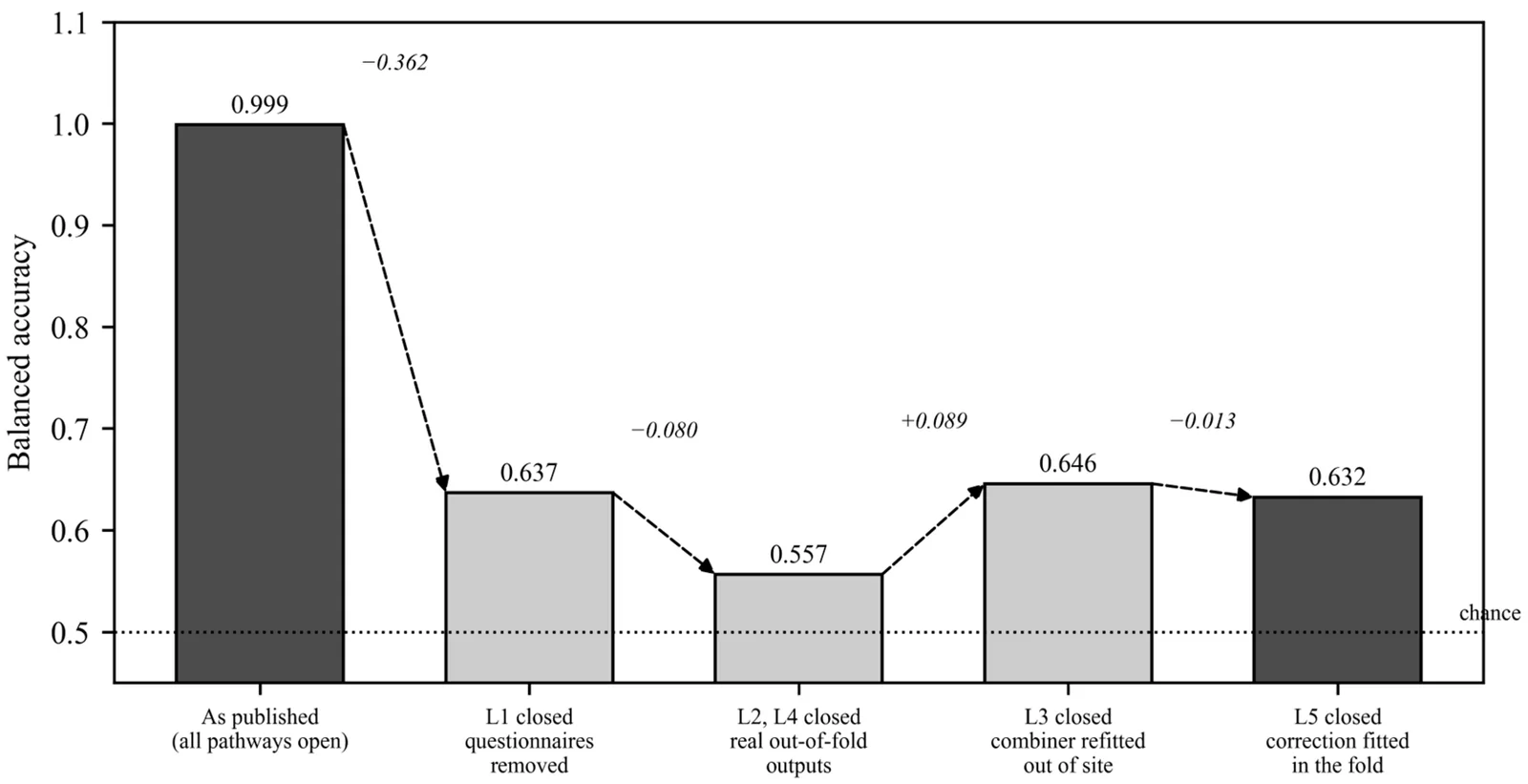
What each leakage pathway was worth. The first bar is the audited pipeline’s reported figure, reproduced from its code; the last is what the same data support when the held-out site touches nothing.

**Figure 6:**
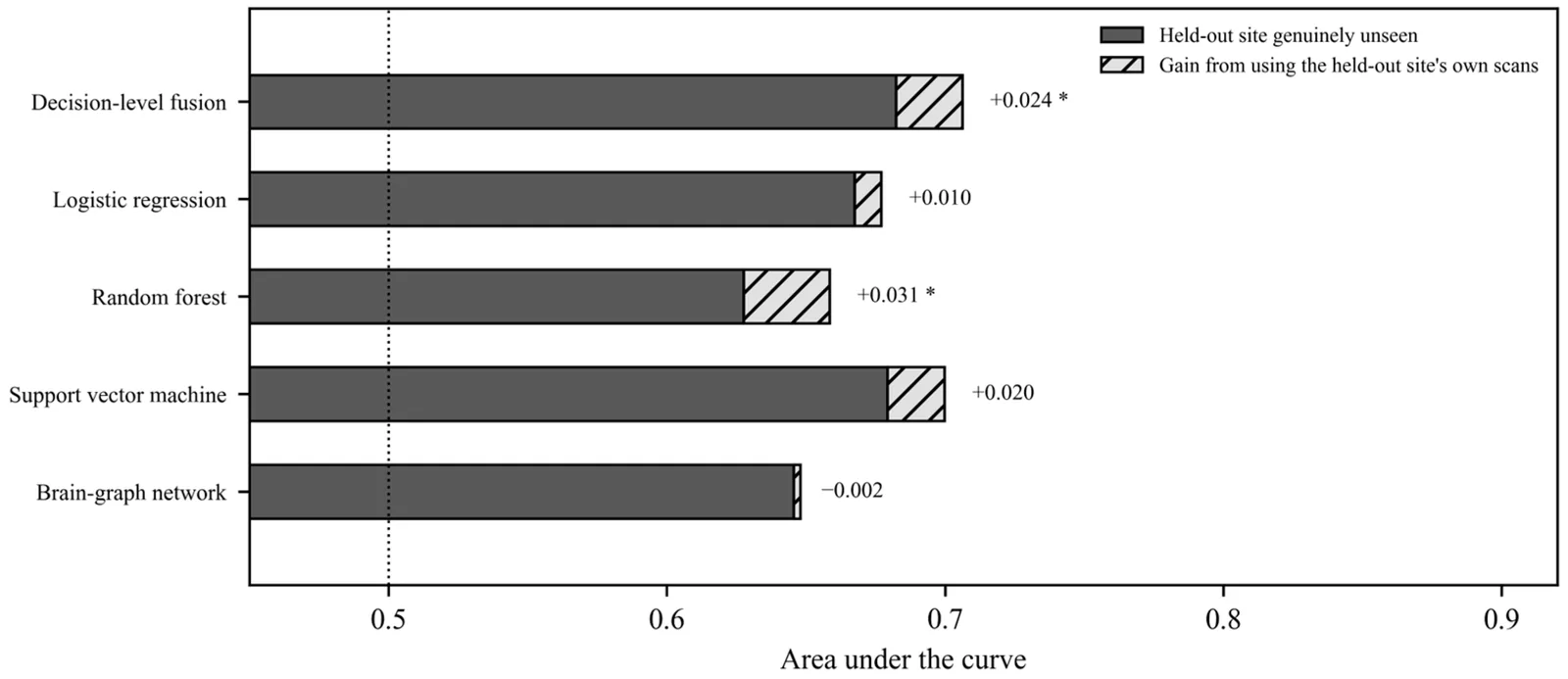
The value of the transductive site correction. The solid bar is strict; the hatched extension is the gain from using the held-out site’s own unlabeled scans. An asterisk marks a gain significant at five percent. The phenotype model, which never sees imaging, gains nothing. The asterisk marks the fusion and random-forest models specifically (Table 7); the kernel machine’s gain is in the same direction, but its interval contains zero.

**Figure 7:**
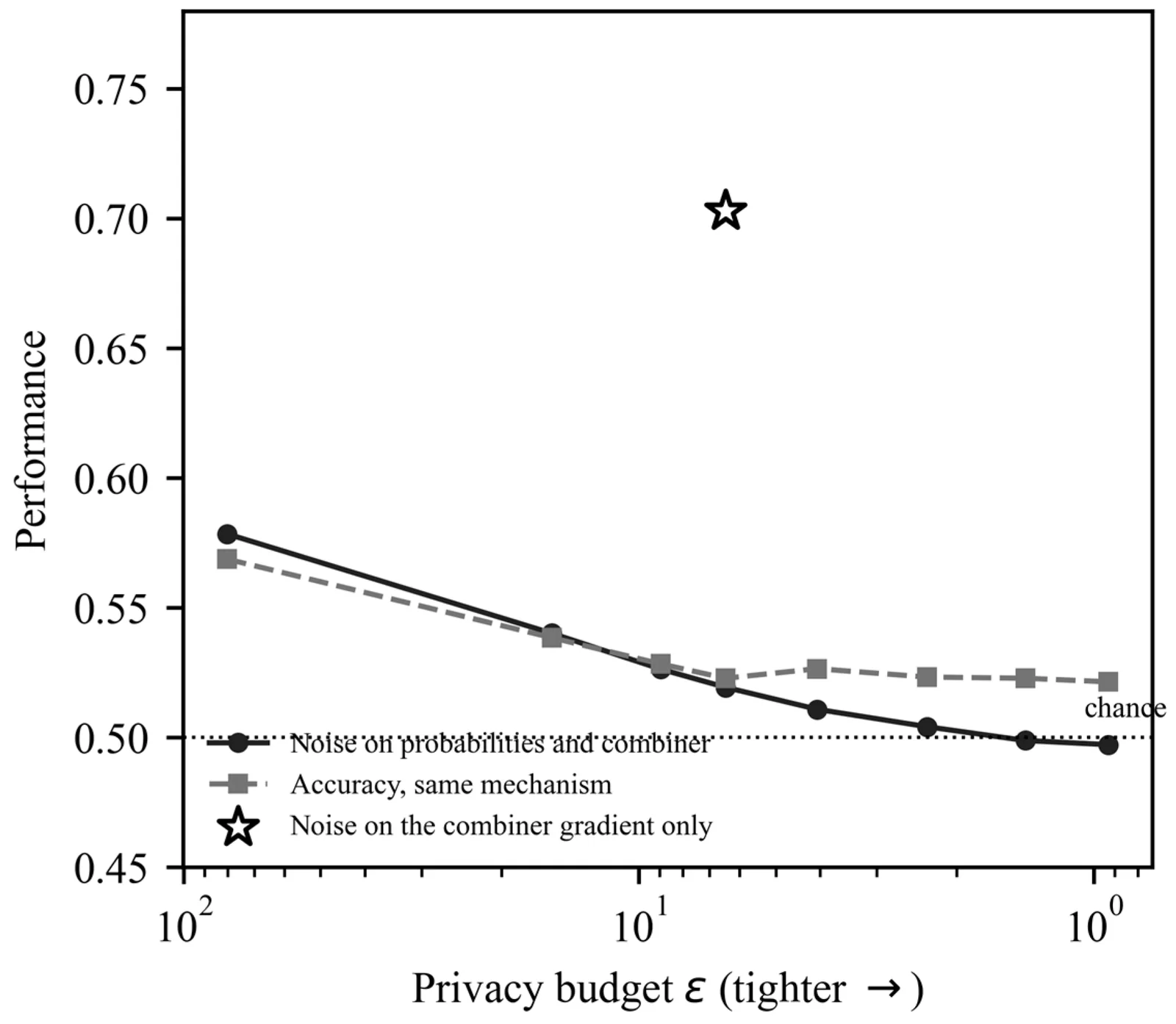
Privacy against utility. Perturbing both the transmitted probabilities and the combiner gradients degrades to chance as the budget tightens. The star marks the mechanism usually reported at the same budget noise on the two combiner weights alone, which appears free.

**Figure 8:**
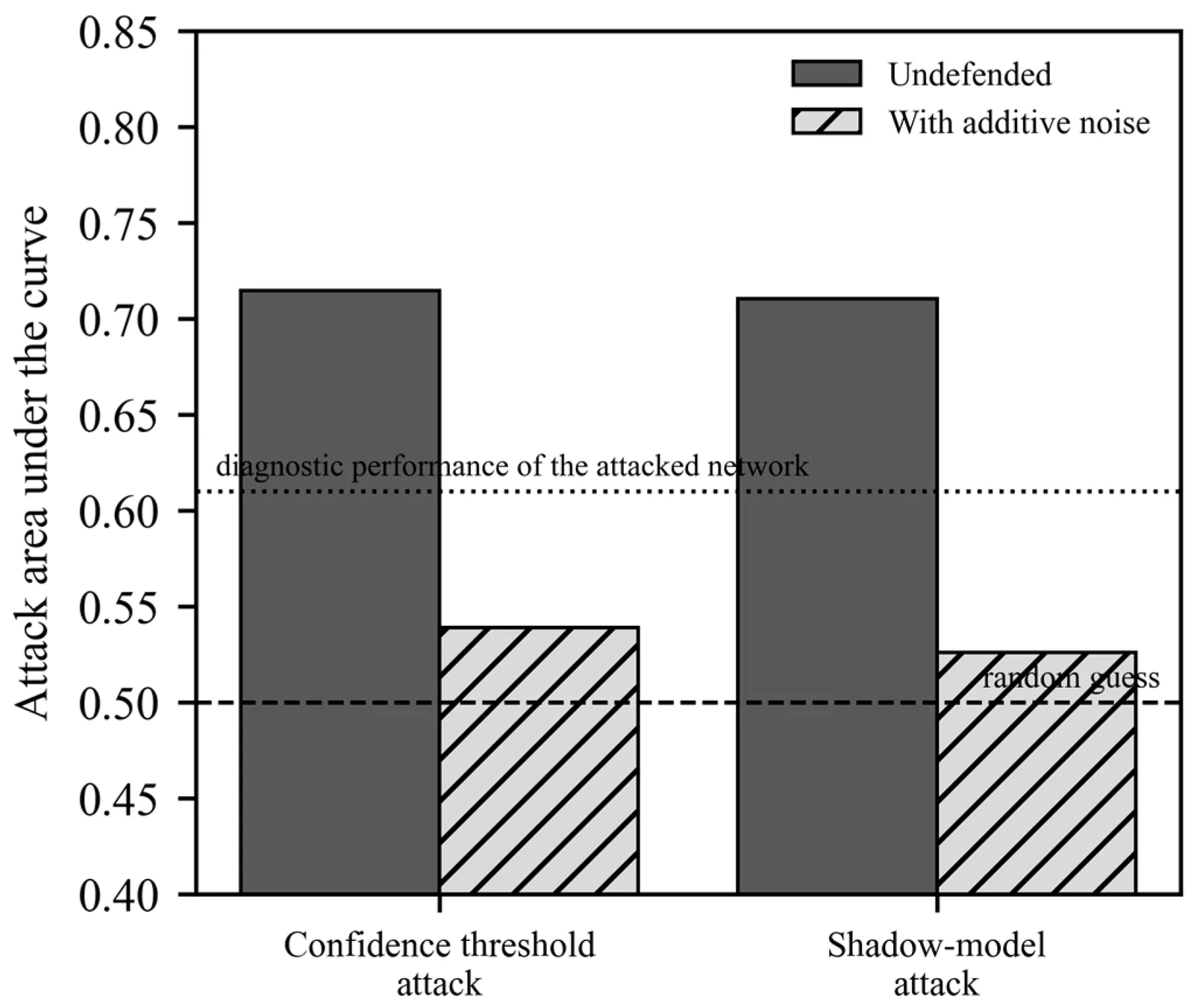
Membership inference. Both attacks on the undefended network exceed its own diagnostic performance (dotted): it memorizes who it was trained on more reliably than it detects the disorder.

**Figure 9:**
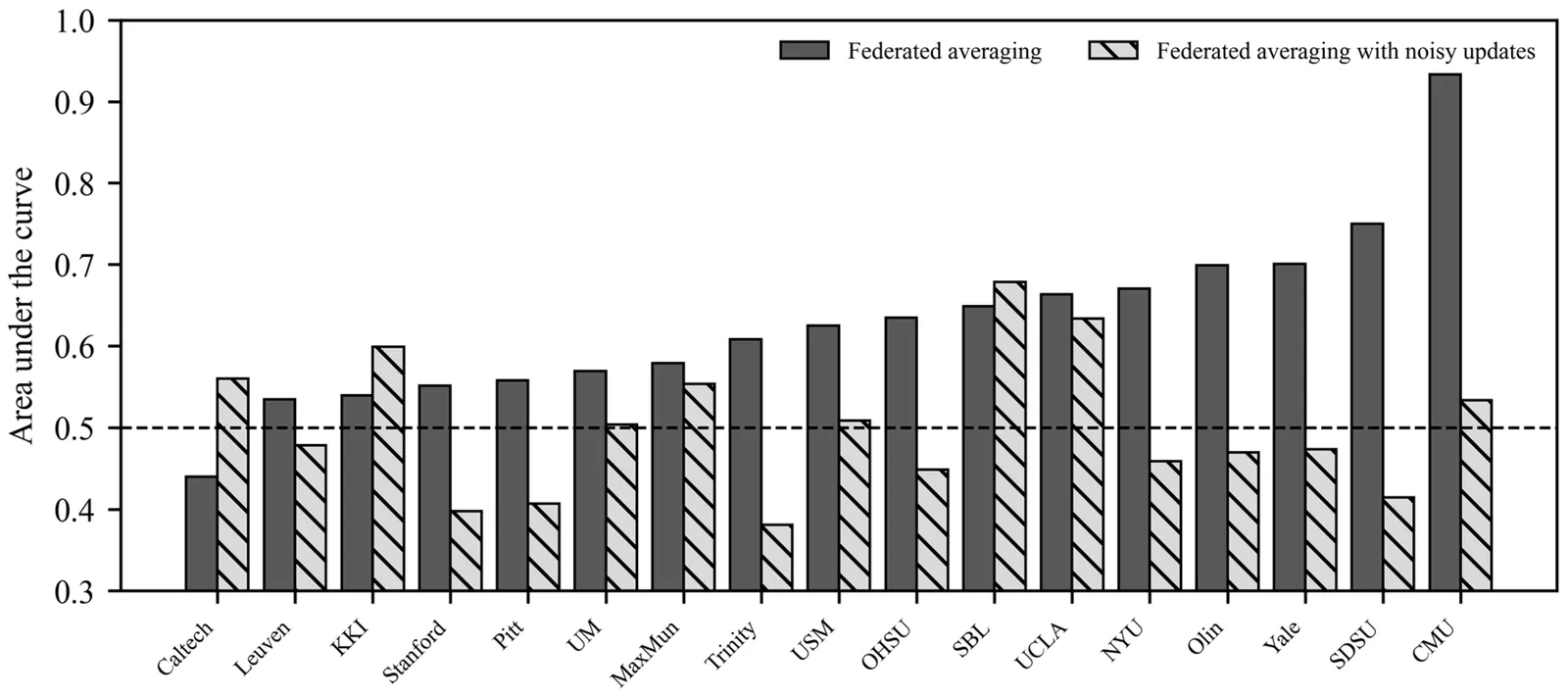
Federated averaging by held-out site. Noisy updates (hatched) collapse the model to chance at almost every site while buying a privacy budget of roughly sixty. The privacy budget (ε ≈ 58) is the Rényi differential-privacy accountant’s value for the noise multiplier used here (Table 10, Section 2.8); an ε this large certifies almost no protection, consistent with the collapse to chance shown.

**Figure 10:**
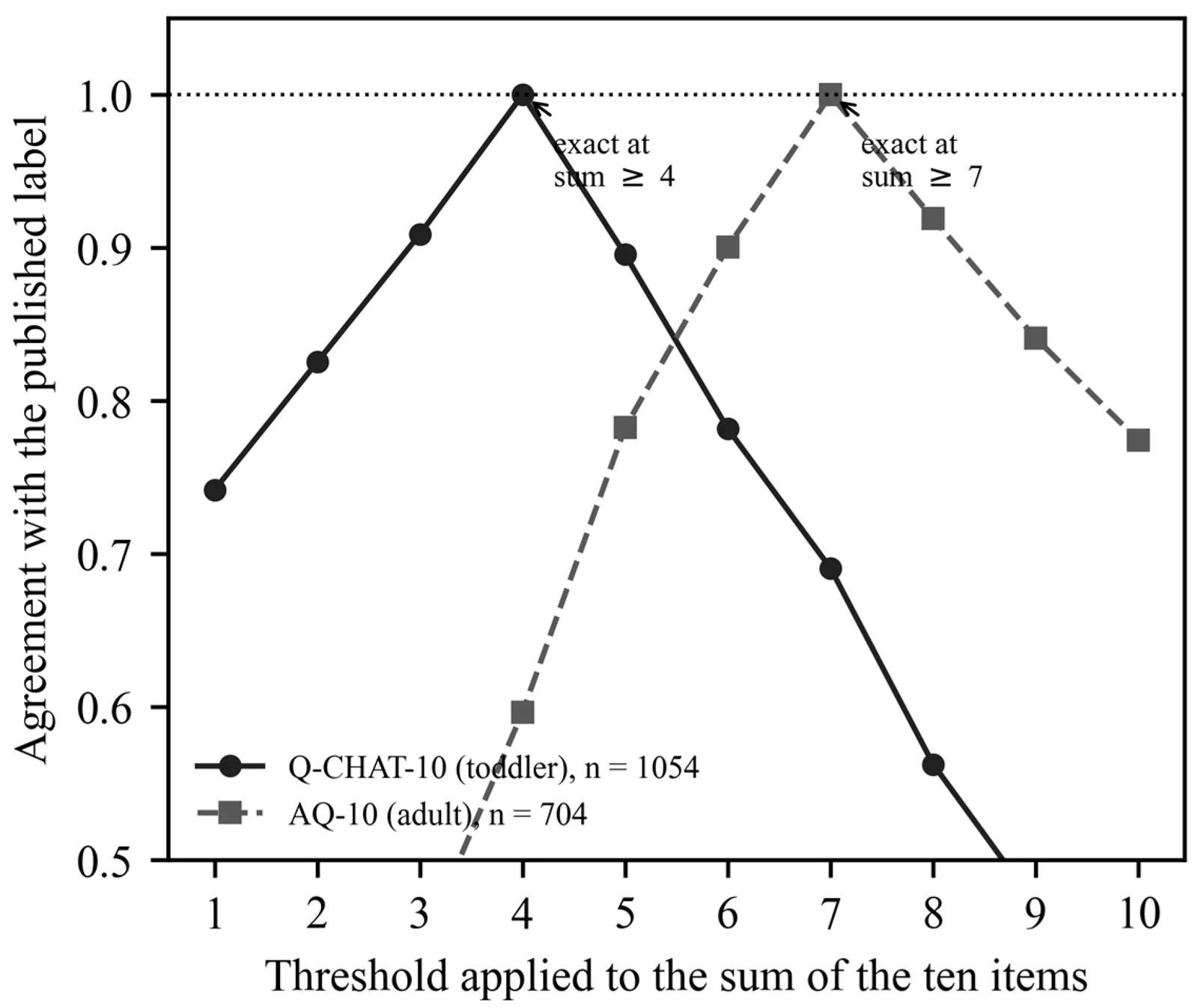
Agreement between the published label and a threshold on the item sum, at every threshold. Both curves touch one: the label is a deterministic function of the classifier’s input.

**Figure 11:**
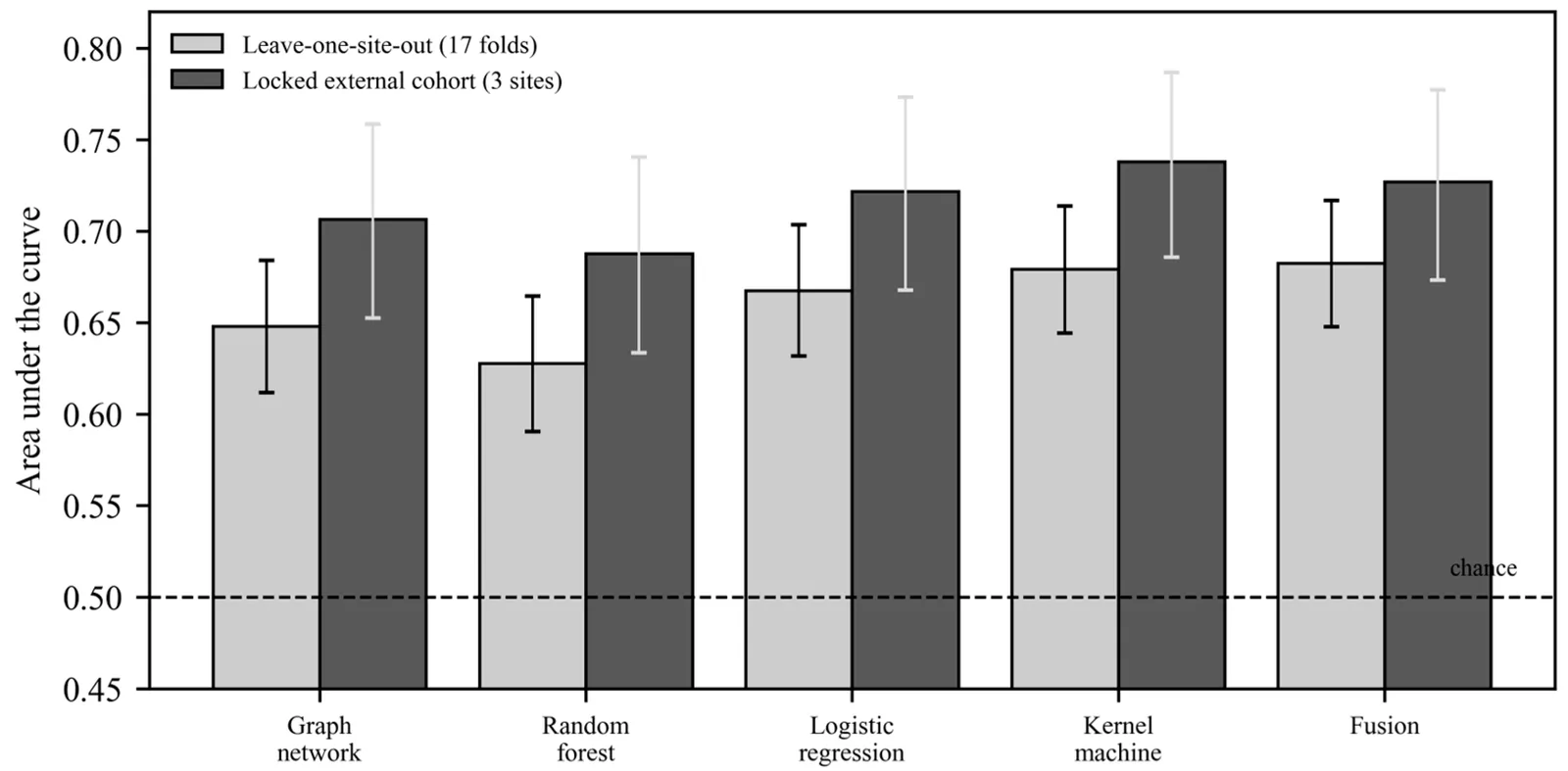
External-cohort validation. Light bars are leave-one-site-out; dark bars are the locked external cohort (359 participants), on which no model was trained. Performance is maintained and the ordering unchanged.

**Table 1:** Composition of the imaging cohort after quality control, ordered by sample size.

Site	Participants	Autism Spectrum Disorder	Typically Developing
NYU	172	74	98
UM	120	47	73
UCLA	85	48	37
USM	67	43	24
Leuven	56	26	30
Pitt	50	24	26
MaxMun	46	19	27
Trinity	44	19	25
Yale	41	22	19
KKI	33	12	21
Olin	28	14	14
SDSU	27	8	19
SBL	26	12	14
OHSU	25	12	13
Stanford	25	12	13
Caltech	15	5	10
CMU	11	6	5
All sites	871	403	468

**Table 2:** The five leakage pathways closed in this work, and the change made. Section 3.4 prices each; Section 3.5 measures the fifth.

Pathway	What the Audited Implementation Did	This Work
L1 tautological label	Both questionnaires used as classification sites	Excluded from every model; audited in Section 3.9
L2 test-set model selection	The checkpoint best on the held-out site was kept	Early stopping on training sites; configuration chosen on separate development sites
L3 in-sample combiner	Fusion weights fitted and scored on the same participants	Weights and threshold refitted per held-out site (Section 3.3)
L4 label-derived probabilities	Probabilities sampled from a Gaussian on the label	All streams are real out-of-fold model outputs
L5 transductive site correction	Correction fitted across all sites, held-out one included	Refitted inside every fold; the held-out site receives none of its own (Section 3.5)

**Table 3:** Performance under the strict protocol, pooled over all 871 participants. Intervals are 10,000-sample bootstraps. Single models are scored at 0.5; the fusion at the threshold of its own fold. *Bold marks the best value in each column.*

Model	Area Under the Curve (95% CI)	Balanced Accuracy	Sensitivity	Specificity	MCC
Brain-graph network	0.648 (0.612–0.684)	0.598	**0.772**	0.425	0.208
BrainGNN-style network	0.675 (0.640–0.711)	0.625	0.692	0.558	0.251
Phenotype model	0.615 (0.578–0.652)	0.600	0.501	0.699	0.204
Random forest	0.628 (0.590–0.664)	0.577	0.417	**0.737**	0.163
Logistic regression	0.667 (0.632–0.704)	0.612	0.591	0.632	0.223
Support vector machine	0.679 (0.644–0.714)	**0.634**	0.600	0.667	**0.267**
Decision-level fusion	**0.682 (0.648–0.717)**	0.632	0.630	0.635	0.264

**Table 4:** Area under the curve for each held-out site, ordered by sample size. The last row is the mean across sites, not the pooled figure of Table 3. *Bold marks the better-performing model at each site.*

Held-Out Site	Participants	Brain-Graph Network	Support Vector Machine	Fusion
NYU	172	0.709	**0.754**	0.716
UM	120	0.710	0.716	**0.741**
UCLA	85	0.738	0.725	**0.766**
USM	67	0.792	0.797	**0.803**
Leuven	56	0.682	0.697	**0.751**
Pitt	50	0.683	0.691	**0.708**
MaxMun	46	**0.558**	0.511	0.519
Trinity	44	0.684	**0.760**	0.758
Yale	41	0.756	**0.768**	0.746
KKI	33	0.512	0.631	**0.754**
Olin	28	0.730	**0.816**	0.806
SDSU	27	0.697	**0.770**	0.704
SBL	26	0.560	**0.661**	0.423
OHSU	25	0.462	0.538	**0.667**
Stanford	25	**0.571**	0.513	0.513
Caltech	15	**0.460**	0.420	0.440
CMU	11	0.800	0.900	**0.933**
Mean	—	0.653	0.686	**0.691**

**Table 5:** Decision-level combiners. The out-of-site column refits weights and threshold per held-out site; the in-sample column fits them once on all participants. The gap is leakage. *Bold marks the best out-of-site area under the curve.*

Combination	AUC (Out-of-Site)	Balanced Accuracy	MCC	AUC (In-Sample)
kernel + demography	**0.682**	0.632	0.264	0.697
graph + kernel + demography	0.682	0.614	0.227	0.700
graph + demography	0.662	0.620	0.239	0.674

**Table 6:** The reported number after each pathway is closed. The first row is the audited pipeline’s own headline, reproduced from its code. The fourth rises because the reported system also changes, not because a leak improves performance. *Bold marks the balanced accuracy this paper reports as its headline result.*

Evaluation	Balanced Accuracy	Change
As published all four pathways open	0.999	—
L1 closed the tautological questionnaires removed	0.637	−0.362
L2 and L4 closed real out-of-fold model outputs, no test-set model selection	0.557	−0.080
L3 closed the combiner refitted out of site, and the best honest system (kernel machine + demography) reported instead of the graph network alone	0.646	+0.089
L5 closed the site correction fitted inside the fold	**0.632**	−0.013

**Table 7:** The value of the fifth pathway. Both columns use identical folds and seeds and differ only in whether the held-out sites own unlabeled scans define its correction. The phenotype model is identical by construction. *Bold marks a gain significant at five percent.*

Model	Held-Out Site Unseen	Own Scans Used	Difference	*p*
Brain-graph network	0.648	0.646	−0.002	0.883
Support vector machine	0.679	0.700	+0.020	0.053
Random forest	0.628	0.658	**+0.031**	**0.019**
Logistic regression	0.667	0.677	+0.010	0.408
Phenotype model	0.615	0.615	+0.000	—
Decision-level fusion	0.682	0.706	**+0.024**	**0.026**

**Table 8:** Privacy against utility. Rows perturb the transmitted probabilities and the combiner gradients; ε is the accountant of Equations (6) and (7) applied to the gradient mechanism, which does not model perturbation of the probability streams.

Noise Multiplier σ	Privacy Budget ε	Accuracy	Area Under the Curve
0.0	∞	0.637	0.690
0.5	80.24	0.569	0.578
1.0	15.50	0.538	0.540
1.5	8.93	0.528	0.526
2.0	6.45	0.523	0.519
3.0	4.05	0.527	0.511
5.0	2.32	0.523	0.504
8.0	1.41	0.523	0.499
12.0	0.93	0.521	0.497

**Table 9:** Membership inference against a network trained on a stratified half of the cohort. 0.5 is perfect privacy. The last rows are the same network’s own behavior on its held-out half.

Quantity	Undefended	With Additive Noise (σ = 2)
Confidence-thresholding attack	0.715	0.539
Shadow-model attack (ten shadows)	0.710	0.526
Training–test accuracy gap	0.305	—
Diagnostic area under the curve	0.610	—

**Table 10:** Federated averaging across the seventeen sites, thirty rounds of two local epochs. The budget is optimistic for the reason in Section 2.8.

Configuration	Mean Accuracy	Mean Balanced Accuracy	Mean AUC	ε
Federated averaging	0.591	0.587	0.630	∞
With noisy updates (σ = 0.8)	0.503	0.495	0.500	58

**Table 11:** Agreement between the published label and a threshold on the sum of the ten items. At the recovered rule, the agreement is exact, so the label carries no information that the items do not.

Dataset	Records	Rule Recovered	Agreement	Best Other Rule
Q-CHAT-10 (toddler)	1054	sum ≥ 4	100.00%	90.89%
AQ-10 (adult)	704	sum ≥ 7	100.00%	91.90%

**Table 12:** External-cohort validation. Every model is trained, selected, and thresholded on fourteen sites and evaluated once on the three largest (359 participants). The leave-one-site-out column is Table 3, for comparison.

Model	External AUC (95% CI)	Balanced Accuracy	MCC	Leave-One-Site-Out
Brain-graph network	0.706 (0.652–0.758)	0.644	0.304	0.648
Phenotype model	0.622 (0.563–0.681)	0.601	0.203	0.615
Random forest	0.688 (0.633–0.741)	0.625	0.250	0.628
Logistic regression	0.722 (0.668–0.773)	0.640	0.280	0.667
Support vector machine	0.738 (0.686–0.787)	0.669	0.338	0.679
Decision-level fusion	0.727 (0.673–0.777)	0.637	0.274	0.682

## Data Availability

All model predictions, trained artifacts, and analysis code are openly available in Zenodo at https://doi.org/10.5281/zenodo.21411486. The imaging data were derived from a public-domain resource, the Autism Brain Imaging Data Exchange (ABIDE-I), http://preprocessed-connectomes-project.org/abide/, and are not redistributed here.
